# The association between reproductive health smartphone applications and fertility knowledge of Australian women

**DOI:** 10.1186/s12905-020-00912-y

**Published:** 2020-03-04

**Authors:** Emmalee A. Ford, Shaun D. Roman, Eileen A. McLaughlin, Emma L. Beckett, Jessie M. Sutherland

**Affiliations:** 1 Priority Research Centre for Reproductive Science, Schools of Biomedical Science & Pharmacy and Environmental & Life Sciences, University of Newcastle, Ring Road, Callaghan, New South Wales 2308 Australia; 2 Hunter Medical Research Institute, New Lambton Heights, New South Wales 2305 Australia; 3 Priority Research Centre for Drug Development, University of Newcastle, Callaghan, New South Wales 2308 Australia; 4 School of Science, Western Sydney University, Penrith, NSW 2751 Australia; 5 School of Biological Sciences, Faculty of Science, University of Auckland, Auckland, 1142 New Zealand; 6 School of Environmental & Life Sciences, Faculty of Science, University of Newcastle, Callaghan, New South Wales 2308 Australia

**Keywords:** Female infertility, Education, mHealth, Fertility awareness, Menstrual cycle

## Abstract

**Background:**

Previous studies have identified that women living in developed countries have insufficient knowledge of factors which may be contributing to the increasingly high global infertility rates such as maternal age and assisted reproductive technologies. There is a large market of reproductive health smartphone applications, yet little is known about the advantages these apps may confer to users in regards to reproductive health knowledge.

**Methods:**

An anonymous, online survey of women living in Australia aged 18 and above was open March–June 2018, until ≥200 responses were acquired for statistical power. Respondents answered questions regarding knowledge about general fertility and related factors (age, cyclic fertility, smoking, obesity, miscarriage rate, and success of assisted reproductive technologies). Fertility knowledge was compared in respondents who did or did not use apps relating to female reproductive health. Additionally the functions preferred in reproductive health apps was described by app using respondents. Sociodemographic information was also collected, and relevant data within the dataset was subject to multivariable modelling for the outcome of the fertility knowledge questions.

**Results:**

Of the 673 respondents that completed the survey, 43.09% reported using mobile phone applications relating to female reproductive health. On average, respondents answered only three of the six fertility knowledge questions correctly. App using respondents were more likely to score better on one question, related to fertility during the menstrual cycle (*p* < 0.001). App users most commonly reported using the menstrual tracking function in apps (82.4%), which may account for the increased knowledge of cyclic fertility.

**Conclusions:**

This data provides preliminary evidence toward the usefulness of smartphone applications as a medium for providing information about fertility to women. A limited understanding of one’s own fertility was demonstrated despite being essential for the decision-making of women throughout their reproductive years.

## Background

Human infertility is recognised as a global public health issue by the World Health Organisation, [1, 2]. Importantly, multiple modifiable lifestyle factors, including smoking and obesity, are known to be detrimental to fertility [3, 4] and the success of reproductive treatment outcomes [5, 6]. However, the age-related fertility decline remains the single most limiting factor in reproductive success [7, 8], with advanced maternal age (> 35 years) associated with an increased risk of infertility, miscarriage, fetal abnormalities, and stillbirth [9–11]. Despite these considerable health risks almost one-quarter of all women giving birth in Australia in 2016 were > 35 years of age [12]. Moreover, within Australian and New Zealand, for 61% of all couples accessing assisted reproductive treatment cycles in 2016, the female partner seeking treatment was > 35 years of age [12].

Although a well-established concept, the consequences of advanced maternal age on fertility and pregnancy may remain poorly understood among the general public. A series of recent international studies have demonstrated that women of reproductive age from Western and European societies consistently underestimate the impacts of maternal age on fertility [13–18]. Additionally, there is a common misconception within Western societies that assisted reproductive treatments can effectively compensate for age-related infertility [19]. These studies support the notion that insufficient knowledge of these factors may be contributing to the number of people struggling with infertility. This data highlights a requisite need for further public education on the consequences of advanced maternal age on fertility and pregnancy for women during their reproductive years.

The United Nations Educational, Scientific and Cultural Organisation recommends that sexual health education include fertility; however this is not currently mandated within many national curricula, including Australia and Britian [20–22]. Despite this, there is an abundance of publicly available information regarding fertility and reproductive health, with many people preferencing the internet to access this content [23, 24]. However, studies demonstrate that people accessing health-related information online are reluctant to go beyond the first page of search engine inquiries, their evaluative skills are limited, and indicators of credibility are often missed (reviewed in [25]). The development of strategies for optimising accessibility and visibility of fertility information is a valuable avenue to improve knowledge.

The global expansion in mobile education products [26] presents the ideal platform for mobile health (mHealth) smartphone applications (apps) to address this knowledge gap. Smartphones are arguably the most accessible form of mixed-modal communication today, with higher online growth than personal computers [27]. In Australia, 84% of all adults, and 99% of 18–29 year-olds possess a smartphone [28]. Demand for mHealth services is demonstrated by the millions of annual downloads across the hundreds of thousands of health and lifestyle apps available [29]. Women’s reproductive health apps account for 7% of all health-related apps [30, 31]. Despite containing a myriad of accessible functions including menstrual cycle tracking, pregnancy planning, and contraception, it remains unknown how these functions influence the fertility knowledge of users. Harnessing these technologies to disemminate reliable and accessible information requires improved understanding of the relationship between their use and the acquisition of relevant knowledge.

Regarding the reliability of reproductive health apps, studies report that only 20% are of good quality [30, 32]. It remains unclear as to whether currently available apps confer understanding, with previous studies of fertility knowledge in women lacking any reference to apps [17, 18, 23, 33–36]. The aim of this study was to determine the differences between fertility knowledge, based on the use of female reproductive health apps, via an anonymous online survey of Australian women. It was hypothesised that women who utilised reproductive health apps would perform better in general fertility knowledge questions Additionally, for women using apps, determining the reproductive health functions employed was a secondary aim. A better understanding of these relationships may reveal new opportunities for the integration of public health interventions within reproductive health apps as a strategy to improve fertility knowledge.

## Methods

### Study participants

This study was approved by the Human Research Ethics Administration at the University of Newcastle; protocol number H-2018-0014. Participation in the survey was limited to women (and for inclusivity, to those not identifying as women, but assigned female at birth), living in Australia, aged 18 and over. Exlcusion from participation was restricted to people with male-assigned reproductive systems, aged under 18 years, or not living within Australia. The use of “female reproductive health” or simply “reproductive health” in this paper refers to aspects surrounding general functions of the female reproductive system including menstruation, conception, and pregnancy.

### Survey design

An anonymous, online fertility knowledge survey was created to capture the usage habits of those with reproductive health smartphone applications (apps), and what relationships exist between differences in knowledge or function of these tools (see supplementary file 1). All respondents provided mandatory information regarding sociodemographic factors including age, education level, income, postcode, relationship status, and pregnancy history. Respondents also completed six, multiple-choice general fertility knowledge questions (supplementary figure 1). These questions covered; fertility during the menstrual cycle, age-related fertility, impact of lifestyle factors (obesity, cigarette smoking) on fertility, the frequency of miscarriage in Australia, and the success rates of assisted reproductive technologies in Australia. These questions were chosen to capture a range of factors relating to aspects of fertility and education including personal management (cyclic fertility and age), lifestyle factors (smoking, obesity), and common misconceptions (miscarriage rate and IVF success). Similar questions have been included in other general fertility knowledge surveys [17, 18, 37, 38] and thus serve the additional purpose of contextualising the results of this study. For respondents using reproductive health apps, the functions and utility preferences of the apps were collected. Reproductive health apps were defined to respondents as “applications that have to do with the female reproductive system and may include features like: menstruation tracking/calendar, pregnancy, or contraception/birth control.”

The survey was implemented using the host website eSurvey Creator (enuvo GmbH, Zurich).

### Data collection

The survey was open in March – June 2018, and advertised on social media (Twitter, Facebook, Instagram), and at the University of Newcastle’s Callaghan, Ourimbah and New Space campuses.

The responses to fertility knowledge multiple-choice questions were coded into a binary of being correct or incorrect. Area data was provided in the survey as the respondents’ postcodes and was re-coded according to the Accessibility/Remoteness Index of Australia (ARIA), which defines remoteness as accessibility based on road distances [39]. In this study, locations with ARIA service centre score ‘A’ were re-coded as “city”, score ‘B’ as “inner regional”, and score ‘C’ as “outer regional”. Annual household income data was provided as a multiple choice of different income brackets. In this study, income data were presented alongside government definitions of low, middle and high income households according to equivalised disposable household income estimates within given quintiles of the population [40]. These estimates are adjusted by equivalence factors to standardise them for variations in household size and composition, while taking into account the economies of scale that arise from the sharing of dwellings [40].

App users were asked about function and utility preferences for their reproductive health apps in a multiple response style question, with an additional free-text response option. Thus, within a given category of this question, the number of responses refers to the proportion of all app using respondents selecting the given category, with the free text option re-coded as “other”.

### Statistical analysis

It was determined prior to launch that the survey required a minimum of 200 responses to allow the detection of a quantitative difference mean response of experimental (app users) and control subjects (non-app users) with probability (power) 0.8 at 0.05 significance level, assuming equal response rates between users and non-users of the apps in question. Data from the survey were entered in JMP version 13 (SAS Institute, Inc.).

Frequencies and proportions were used to describe the range of categorical responses, and comparisons of proportions between app users and non-app users were made by chi-square likelihood ratio tests, with effect sizes reported using the r-squared statistic. *P* < 0.05 was considered to be statistically significant. Where the proportions of a sociodemographic category differed significantly between app users and non-app users, the category was then used as a predictor in multivariable models for the outcome of the fertility knowledge questions.

Nominal logistic regression was used for multivariable models. The chi-square statistic for the whole model was reported, with adjustments for the following variables: age, app use, whether the respondent had conceived in the past, and if they were currently trying to conceive. Effect likelihood ratio tests, odds ratios, and 95% confidence intervals were reported for predictors.

## Results

### Sociodemographic and reproductive history of respondents

At the end of the survey period a total of 759 respondents had accessed the online survey, with 673 respondents completing all mandatory items allowing their inclusion in data analyses. A majority of the respondents were of reproductive age (between 18 and 36 years of age), making up 83.8% of all respondents (Table 1). Those who had completed a tertiary degree (or above) comprised 43.3% of all respondents (Table 1). Of the 227 respondents who had conceived, 78.4% (178) had given birth. The current use of smartphone apps relating to reproductive health was reported by 43.1% (290) of respondents. App users were as a group younger than non-app users (Table 1; χ^2^ = 24.5, *p* < 0.001). A larger proportion of app users lived outside of metro areas, in inner or outer regional areas (27.9% vs 18.3%; χ^2^ = 8.8, *p* = 0.013). Additionally, it was more likely that app users were trying to conceive (8.3% vs 1.6; χ^2^ = 18.1, *p* < 0.001), and were more likely to have previously conceived compared to non-app users (39.3% vs 29.55%; χ^2^ = 7.1, *p* = 0.008).

**Table 1:** Sociodemographic and reproductive history of respondents sorted by reproductive health application usage status

	**All respondents (*n*** **= 673)**	**App-users (*n*** **= 290)**	**Non-app users (*n*** **= 383)**	**Χ**^**2**^**(*P*-value)**^**a**^
Age category	*n* (%)	*n* (%)	*n* (%)	
18–24	285 (42.4)	130 (44.9)	155 (40.5)	24.540 (< 0.0001^c^)
25–30	161 (23.9)	71 (24.5)	90 (23.5)	
31–36	118 (17.5)	59 (20.3)	59 (15.4)	
37–42	46 (6.8)	20 (6.9)	26 (6.8)	
43+	63 (9.4)	10 (3.5)	53 (13.9)	
Location by ARIA category				
City	522 (77.6)	209 (72.1)	313 (81.7)	8.769 (0.0125^c^)
Inner regional	138 (20.5	74 (25.5)	64 (16.7)	
Outer	13 (1.9)	7 (2.4)	6 (1.6)	
Education attained				
< Year 12 (or equivalent)	25 (3.7)	10 (3.5)	15 (3.9)	5.261 (0.2615)
Year 12 (or equivalent)	157 (23.3)	80 (27.6)	77 (20.1)	
Technical diploma	109 (16.2)	43 (12.9)	66 (17.2)	
Bachelor’s degree	256 (38)	106 (36.5)	150 (39.2)	
Postgraduate	126 (18.7)	51 (17.6)	75 (19.6)	
Annual household income				
< $20,000	78 (11.6)	34 (11.7)	44 (11.5)	11.511 (0.0738)
$20,000 - $34,999: low income^b^	74 (11)	32 (11)	42 (10.9)	
$35,000 - $49,999: mid income^b^	67 (10)	25 (8.6)	42 (10.9)	
$50,000 - $74,999	109 (16.2)	41 (14.1)	68 (17.8)	
$75,000 - $99,000	98 (14.6)	53 (18.3)	45 (11.8)	
$100,000 - $149,999: high income^b^	141 (21)	68 (23.5)	73 (19)	
$150,000 +	106 (15.8)	37 (12.8)	69 (18)	
Relationship status				
Married/de facto	358 (53.1)	158 (54.5)	200 (52.2)	1.7 (0.6368)
In a relationship, living apart	133 (19.8)	55 (19)	78 (20.4)	
Not in a relationship	170 (25.3)	70 (24.1)	100 (26.1)	
Other/not disclosed	12 (1.8)	7 (2.4)	5 (1.3)	
Have a close friend/relative who has experienced fertility issues				
Yes	381 (56.6)	156 (53.8)	225 (58.8)	1.647 (0.1993)
No	292 (43.4)	134 (46.2)	158 (41.3)	
Currently trying to conceive				
Yes	30 (4.4)	24 (8.3)	6 (1.6)	18.052 (0.0001^c^)
No	639 (95)	264 (91)	375 (97.9)	
Not disclosed	4 (0.6)	2 (0.7)	2 (0.5)	
Have conceived before				
Yes	227 (33.7)	114 (39.3)	113 (29.5)	7.073 (0.0078^c^)
No	446 (66.3)	176 (60.7)	270 (70.5)	
Have given birth				
Yes	178 (26.5)	87 (30)	91 (23.7)	3.287 (0.0698)
No	495 (73.5)	203 (70)	292 (76.3)	

^a^Indicates that statistical data derived from comparison between app users and non-app users

^b^Reference to Australian Bureau of Statistics definitions of household wealth [40]

^c^Indicates statistical significance

### General fertility knowledge of respondents

**Fig. 1:**
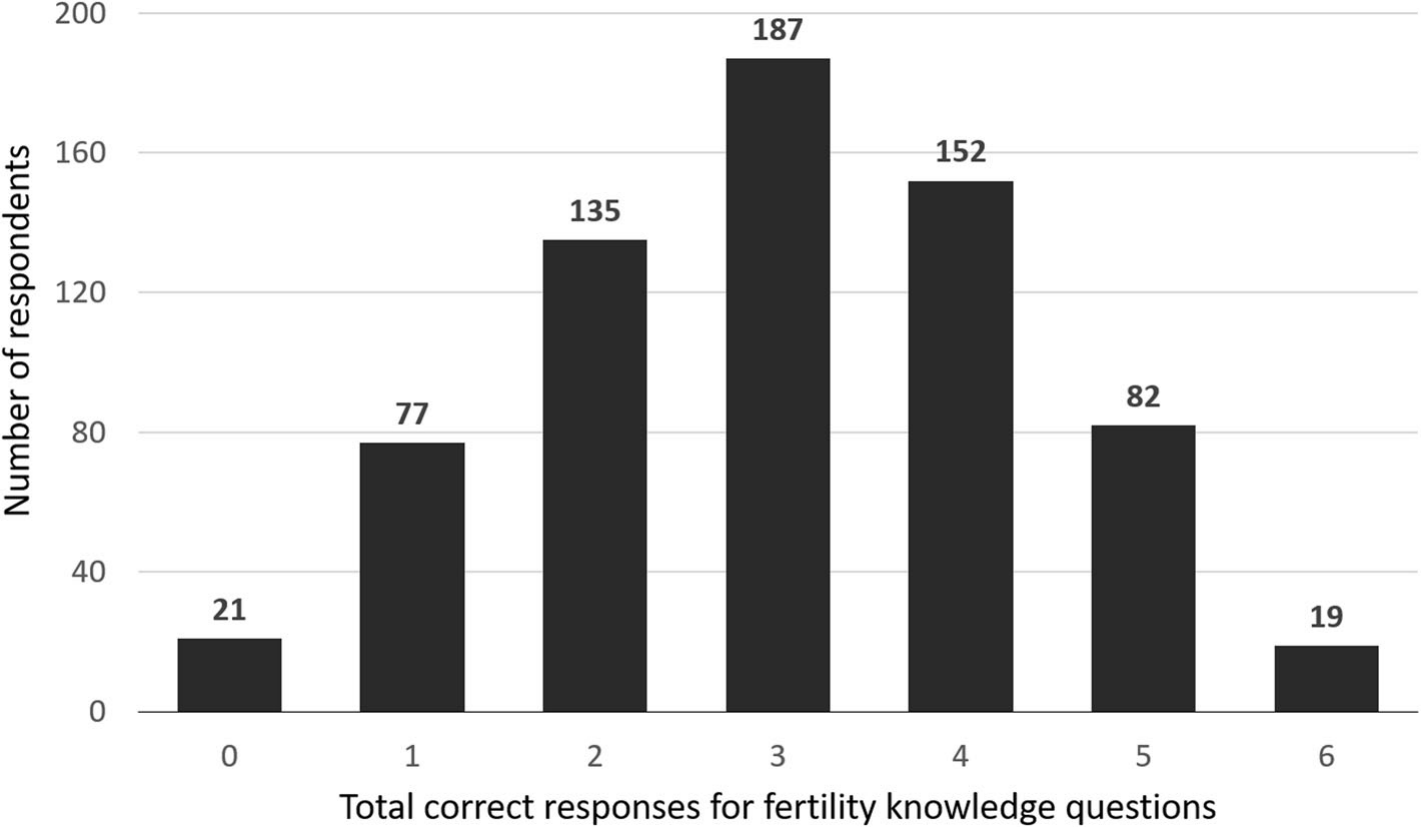
Total score for fertility knowledge questions. In the fertility knowledge survey, respondents were asked six general knowledge fertility questions (Supplementary figure 1) with multiple choice responses, and the total score of correct responses for each respondent across the questions is represented in the graph (*n* = 673)

The cumulative scores for the six multiple-choice fertility knowledge questions are shown in Fig. 1. The data follows a Poisson distribution (χ^2^ goodness of fit = 423.1, *p* = 1). The mean score was 3.03 correct answers, with a standard deviation of 1.38. The modal score was three correct answers, (27.8% of respondents; 95% CI: 24.4–31.2). Surprisingly 3.1% of respondents did not answer any questions correctly, with 31.5% getting only 2 or fewer questions correct. Only 2.8% of respondents answered all six questions correctly.

**Fig. 2:**
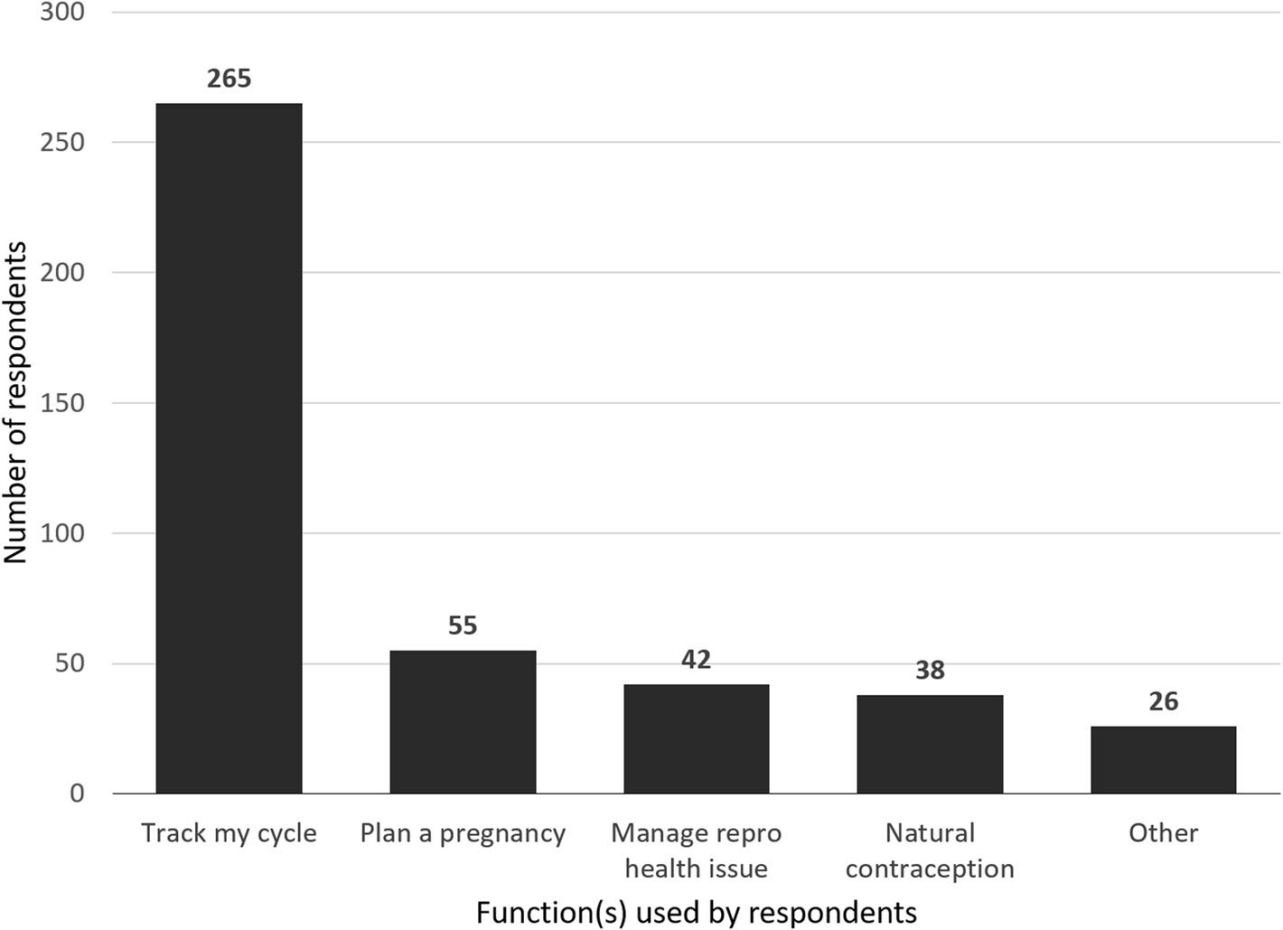
Application user functions and utility preferences. Respondents who use reproductive health applications were instructed to select all that apply from a list of common utilities and functions available in reproductive health smartphones, with a free text option to add other options. Additional functions that respondents commonly identified in ‘other’ included reminders to take birth control [15], and monitoring cycle-related symptoms [9]. Data is presented as a count of the respondents that selected the corresponding option (*n* = 290)

A clear majority of app users tracked their cycles (91.4%, or 265 out of 290 app users), with the next most selected function “plan a pregnancy” accounting for only 19%, or 55 responses (see Fig. 2). Fifteen respondents utilised the free-text tool enabled for ‘other’ to identify that birth control reminders were a primary function they used reproductive health apps. A further 9 items within the ‘other’ category were identified by respondents who use reproductive health apps for monitoring cycle-related symptoms (i.e. headaches, bloating, and acne).

### Comparisons between reproductive health app and non-app users

The proportion of correct responses for each category are shown in Table 2. App users were more likely than non-app users to correctly identify the most fertile time in the menstrual cycle (15.7% χ^2^ = 16.7, *p* < 0.001). These results remained significant in the fully adjusted model (χ^2^ = 44.4, *p* < 0.001). The parameters responsible for this effect were app use (χ^2^ = 16.4, *p* < 0.001) and age (χ^2^ = 10.1, *p* = 0.039). For this question, app users were 1.9 times more likely to answer correctly (95% CI: 1.4–2.8, *p* < 0.001) than non-app users.

**Table 2:** Proportions of correct responses by question

Question	All respondents correct (%)	App users correct (%)	Non-app users correct (%)	***P***-value^**a**^	***P***-value for adjusted model^**c**^
When is the most fertile time in the menstrual cycle?	58.8	64.8	49.1	< 0.0001^b^	< 0.0001^b^
At what age does a woman’s fertility begin to decline?	46.2	42.8	45.2	0.5326	0.1220
How much does cigarette smoking negatively impact fertility?	61.9	57.6	60.3	0.4762	0.7137
How much does obesity negatively impact female fertility?	65.5	60	64.5	0.2336	0.1885
On average, how frequently do you think miscarriages occur in Australia?	50.7	53.4	45.4	0.0726	< 0.0001^b^
On average, how often do you think IVF achieves a live birth in Australia?	34.5	31.4	34.2	0.4398	0.2025

^a^indicates that statistical data derived from comparison between app users and non-app users

^b^Indicates statistical significance

^c^model is adjusted for the following predictors: app use, age, location, whether respondent was trying to conceive, and whether they had conceived before

For the question regarding miscarriage rate in Australia, there were no significant differences in the correct responses of app users compared to non-app users (χ^2^ = 3.22, *p* = 0.07). However, the adjusted model for this question was significantly different (χ^2^ = 44.4, *p* < 0.001), with the effects primarily driven by whether the respondent was trying to conceive (χ^2^ = 24.1, *p* < 0.001), whether they had conceived before (χ^2^ = 14.7, *p* < 0.001), and age (χ^2^ = 11.7, *p* = 0.02). The same adjustments were applied to each subsequent fertility knowledge question, yet there were no significant differences. For the remainder of the questions, there were no statistically significant differences between app users and non-app users.

## Discussion

There is an increasingly large volume of users accessing the range of health apps for female reproductive health. Yet these types of apps have previously only been studied in small samples with a focus on reviewing the information within apps [30, 32, 41, 42], or the design and testing of novel apps [43–47]. Presently, the association between knowledge about female reproductive health and the use of reproductive health-themed apps has yet to be investigated. This study is the first to address the fertility knowledge gap by exploring the relationship between app use and knowledge within a considerably large cohort of Australian women. This study identified a novel association between app use and knowledge of fertility during the menstrual cycle. This association provides compelling preliminary evidence that may be used to assess the viability of apps as a medium to promote public health messages and address the gap in fertility knowledge. This study also found that the most popular function within reproductive health apps was menstrual cycle tracking, making this type of app an ideal opportunity for public health intervention.

A number of demographic variables were different between the app using and non-app using groups in this study. App users were more likely to be younger, aged 18–24, which is expected, as the adoption of smartphones is much greater in this generation [48]. In a 2017 national mobile consumer survey, 95% of 18–34 year olds reported smartphone ownership, compared to older Australians (85% in 45–54 year olds, and this further decreases with advancing age) [48]. A greater proportion of app users lived in regional centres (defined as fewer than 250,000 people), which was an unexpected finding, as regional and rural internet accessibility has traditionally been a challenge for people living away from city centres [49]. However, there is a renewed research focus in the equal distribution of care into rural and region locations in Australia via smartphone technology [50–52], and our findings have positive implications for rural mobile health interventions of the future. Finally, app users were more likely to be trying to conceive, or already had conceived compared to non-app users. This may be attributed to the app functions used by respondents (such as fertility tracking and pregnancy planning), fitting with previous studies which have demonstrated that women who are struggling to conceive often actively try and improve their knowledge through multiple sources [24, 34, 53, 54].

Overall the fertility knowledge of respondents in this survey, regardless of app use, was mediocre, which may suggest that women are lacking education in some fundamental aspects of fertility and reproductive health. This is consistent with the literature surrounding women’s understanding of fertility related to age of fertility decline, cyclic fertility, and lifestyle factors that can influence fertility [13, 15–18, 23, 33, 34, 55]. Misunderstanding aspects relating to fertility may risk women’s future plans of parenthood. In particular, the decision to delay childbearing without an understanding of the negative impact of advanced maternal age on reproductive success [56]. This can ultimately burden the health system, with Australian government-funded health care claims surpassing $200 million for assisted reproductive treatments in 2010 alone [57]. The burden is predicted to rise as the use of assisted reproductive technologies is increasing, while live birth rates as a result of these technologies remains staggeringly low at only 18.1% [12]. Almost two thirds of all respondents overestimated the success rate of IVF. This creates an additional burden as the consequence of delaying conception is not as easily rescued as respondents estimate. Importantly, infertility can have mental health impacts for patients [58, 59], thus the current study only adds to the evidence that more targeted and further reaching public health interventions are required to bridge the fertility knowledge gap. Increasing knowledge about general aspects of fertility and pregnancy may have additional benefits beyond successful conception; it may help adjust women’s expectations and hence their experiences. For instance, amongst women who had experienced a miscarriage, those with more education about miscarriage beforehand were better able to cope with the event when it occurred [60].

The potential response bias occurring due to the large proportion of women in this survey cohort who used menstrual cycle tracking as a function in their reproductive health apps may be a limitation of the present study. The knowledge differences among women who used apps for different reasons was not able to be examined in great depth in this study, thus a larger sample of diverse reproductive health app functions is required in future research. This would enable the link between app function and knowledge improvements to be studied more conclusively. Increasing the specificity of response options available for the ‘track my cycle’ selection in further surveys also may shed more light on this aspect in future research. It is important to note that the proportion of women in the app using cohort that had completed a tertiary degree (or above) is 24% higher than the overall proportion of Australian women with the same qualification at an estimated 30.1%, which needs to be considered in interpretation [61]. In the survey, 24 app using respondents self-identified that they were trying to conceive, yet 55 app using respondents were reportedly using a ‘plan a pregnancy’ app. There are a range of pregnancy-related apps available to accommodate for many stages throughout pregnancy (planning to conceive, trying to conceive, predicting gestation monitoring pregnancy, etc) [42], and respondents may have selected this to cover this broad range of applications. Apart from the free-text option, ‘plan a pregnancy’ was the only selectable option that mentioned pregnancy. Indeed, increasing the specificity of the ranges of reproductive health apps used, or creating further defining questions in futures studies may assist in interpreting any associations between fertility and knowledge.

Prior research has shown that app users of health and medical-type apps [62, 63], and female reproductive health apps [64, 65] often self-report during research interviews the usefulness and ‘benefits’ they obtain from their app use. However, previous research has not quantitatively studied whether these ‘benefits’ are associated with knowledge about the content in question. This is important to consider in order to evaluate the usefulness of these apps as tools for education, to identify the strengths of current apps, and to highlight the challenges for future apps. In this study, it was observed that those who used reproductive health apps were more likely to be knowledgeable of the most fertile time in the menstrual cycle (*p* < 0.0001 in adjusted model). This may be due to the large proportion of app using respondents that utilised menstrual cycle tracking functions. Despite the popularity of menstrual cycle tracking in this study, its use may not necessarily be linked to fertility. A recent study found that women identified many reasons for tracking menstruation using apps, and of the five priorities, only two could be directly connected to providing knowledge about fertility (becoming pregnant, inform conversations with healthcare providers) [64], further description of the purposes for each type of reproductive health app would serve to examine this link more closely. Nevertheless, the fact that 91.4% of women in this study tracked their menstrual cycles using reproductive health apps, provides an excellent intervention opportunity for education regarding cyclic fertility.

While the marketplace of menstrual cycle tracking and other reproductive health apps is fraught with inaccuracies and misinformation [30, 66], there are a small number that meet adequate standards, with a US study finding 18% of the reviewed menstrual cycle tracking apps were accurate [30], and a recent study validating some components of a fertility app [67]. Additionally, around 90% of women have a ‘normal’ cycle frequency (between 24 and 38 days) [68], thus apps that may have flaws in their content relating to those with irregular cycles, may still be beneficial to a majority of users with no cycle irregularity Further research into education gained from apps could involve testing the knowledge of a sample of women before and after prolonged periods using an accurate menstrual tracking application purposed for fertility awareness. Additionally, further analyses linking the quality of apps used by respondents to their demonstration of fertility knowledge obtained from the survey would enable the link between app use and knowledge to be understood in greater detail.

Although not associated directly with app use, an additional and important finding from this study was the observation that women with a history of conception (*p* < 0.001) or who were actively seeing to get pregnant (*p* < 0.001) were more aware of the miscarriage rate (*p* < 0.001 in adjusted model). This is consistent with findings that the general population often underestimate the prevalence of miscarriage [37], and couples who have experienced miscarriage reported that they were not previously aware of the frequency in which it occurs in the people around them [69]. As miscarriage is not often discussed amongst the general population, it is likely that lived experience was responsible for the significant differences in response rates to this question in women who had conceived in the past or that were trying to conceive.

## Conclusions

This study has provided preliminary evidence for an association between fertility knowledge and reproductive health app use. This will be able to inform public health strategies aimed at increasing awareness of fertility throughout a woman’s reproductive years. Fertility knowledge and awareness are important, not only for family planning, but for young people to have realistic expectations and make lifestyle choices accordingly. The association between knowledge and app use in this study while significant, had a small effect size, and covered only one aspect of fertility. This may be a reflection of the disadvantages in the apps themselves as discussed above, or a reflection of the limited knowledge of the general population. In both cases, the distribution of fertility information to women is imperative, and should be delivered in a way that is highly accessible, and likely to be received by women. Smartphone applications may be one such avenue for the dissemination of this information, and this study presents a novel opportunity for public health interventions using this method. Regardless of the sources developing them, mobile phone applications play an increasingly important role in the provision of personal health information [41], and this study provides an insight into how smartphone applications may be able to educate women about their own fertility.

## Supplementary information

**Additional file 1.**

**Additional file 2: Figure S1.** Description of data: Response to fertility knowledge questions by app use status. Each of the questions in the fertility knowledge quiz is displayed with the responses as shown to participants (Δ indicates the correct answer). Black bars represent the number of non-app using respondents (*n* = 383) that selected a given response, and grey bars indicate app users (*n* = 290).

## Data Availability

The datasets generated and/or analysed during the current study are not publicly available due to the likelihood that they will be used in future novel analyses, but are available from the corresponding author on reasonable request.

## Abbreviations

Apps: Smartphone applications
ARIA: Accessibility/Remoteness Index of Australia
IVF: In vitro fertilisation
mHealth: Mobile health
US: United States
