# Supplementary material for: The association between reproductive health smartphone applications and fertility knowledge of Australian women
Source: BMC Womens Health. 2020 Mar 4;20:45. doi: 10.1186/s12905-020-00912-y (PMC7057638; doi:10.1186/s12905-020-00912-y)
Supplement: Supplementary file 1 — Additional file 1. [file 12905_2020_912_MOESM1_ESM.docx]

1. How old are you?
   - 17 and under
   - 18-24
   - 25-30
   - 31-36
   - 37-42
   - 43+
2. What is your postcode? (Australian participants only)
3. What is your highest completed level of education?
   - Below year 12 or equivalent
   - Year 12 or equivalent
   - Technical Diploma (i.e. TAFE)
   - Bachelor’s degree
   - Postgraduate Degree
4. What is your average annual household income?
   - Less than $20,000
   - $20,000 to $34,999
   - $35,000 to $49,999
   - $50,000 to $74-999
   - $75,000 to $99,999
   - $100,000 to $149,999
   - $150,000 or more
5. What is your relationship status? If you feel your relationship isn’t described by the options below, please add your own!
   - Married/de facto relationship
   - In a relationship, but not living together
   - Not currently in a relationship
   - Prefer not to say
   - Other:
6. Have any of your close female relatives had fertility problems (such as trouble conceiving, miscarriage, medical conditions affecting ovaries/uterus?)
   - Yes
   - No
   - Prefer not to say
7. If yes, what was this person’s relationship to you?
   - Me
   - Mother
   - Sister
   - Daughter
   - Aunt
   - Cousin
   - Other:
   - Prefer not to say
8. Are you currently trying to conceive a child?
   - Yes
   - No
   - Prefer not to say
9. Have you conceived in the past?
   - Yes
   - No
   - Prefer not to say
10. Have you given birth?
    - Yes
    - No
    - Prefer not to say
11. Have you ever struggled when trying to conceive (i.e. did it take longer than you originally expected)?
    - Yes
    - No
    - Prefer not to say
12. If yes, how did you overcome this? Select as many that apply or add your own answer
    - Kept trying and eventually fell pregnant
    - Changed lifestyle factors
    - Used assistive reproductive technologies (IVF, ICSI)
    - Stopped trying to conceive
    - Prefer not to say
    - Other:
13. Did you use a reproductive health application before conceiving?
    - Yes, I used it to help me know when in my cycle I was fertile
    - Yes, but I used it for other features as well
    - No
    - Other:
14. Do you use smartphone applications to manage reproductive health? (These apps include, but are not limited to menstrual tracking, pregnancy planners, contraceptive reminders)
    - Yes
    - No
15. If you answered no, tell us why?
    - I haven’t tried it
    - I don’t think it’s relevant to me
    - I used to use one, but now I don’t
    - Other:
16. If you answered yes to question 1, which application do you use (if you use multiple, we would like to know about your most frequently used app)?
    - List of top 10 in app stores, plus “other” box
17. Which application do you use? If you use more than one, we would like to know about the one you use the most
    - Life Period Tracker
    - FMC Ovulation Tracker
    - My Cycles
    - Period Diary
    - Maya: Period, Fertility, Ovulation & Pregnancy
    - Fertility Friend FF App
    - Ovia Fertility Period Tracker
    - Clue
    - Kindara: Fertility Tracker
    - Flo Period & Ovulation Tracker
    - Glow: Best Ovulation Tracker Fertility Calendar App
    - Menstrual Period Tracker
    - Other:
18. What kind of smartphone do you use?
    - iPhone
    - Android
    - Other:
19. What are the main reasons you use this application/s?
    - Track my cycle
    - Natural contraception
    - Plan a pregnancy
    - Manages/records my reproductive health issue (PCOS, Endometriosis, etc)
    - I was recommended by another user
    - I was recommended by health professional
    - Other:
20. What features of this application/s appeal to you? Select as many that apply or add your own
    - Shows me fertile times in my cycle
    - Helps me manage or document my cycle
    - I get to learn more about my reproductive health
    - The app is very easy to use
    - I get reminders on my smartphone
    - My data is secure
    - Other:
21. How long have you been using this application/s?
    - Less than a month
    - 1-3 months
    - 3-6 months
    - 9-12 months
    - 1-2 years
    - 3-4 years
    - 5 or more years
22. How often do you interact with this application/s:
    - A few times a day
    - Once a day
    - A few times a week
    - Once a week
    - Monthly
    - Hardly ever
23. Have you tried other reproductive health-style apps in the past?
    - Yes
    - No
24. If yes, why did you switch? Select as many that apply or add your own
    - Recommended to change to my current app by someone
    - I didn’t like the app I was using at the time
    - I wanted features that the previous app didn’t support
    - Other:
25. How would you rate your own knowledge of the female reproductive system?
    - Very low
    - Low
    - Moderate
    - High
    - Very High
26. Where do you go for information about reproductive health (tick all that apply)
    - Internet
    - Smartphone apps
    - Health professional (GP, gynaecologist)
    - School
    - University
    - Friends
    - family
    - TV
    - Books
    - Magazines
    - Podcasts
    - Other
27. If you ticked more than one option for the previous question, can you tell us about the information you searched for?
    - Same kind of information from each source
    - Different kinds of information depending on which source
    - Tell us more about what kinds of information you looked for indifferent sources:
28. If applicable, how useful to you is the information provided by your reproductive health application(s)?
    - Very useful
    - Useful
    - Somewhat useful
    - Rarely useful
    - Never useful
29. Based on your knowledge, when is the most fertile time in a menstrual cycle?
    - In the middle week between periods
    - In the week just after a period
    - In the week before the next period
    - Almost any day except during a period
    - During a period
    - Don’t know
30. Based on your knowledge, at what age does female fertility begin to decline?
    - Age doesn’t matter
    - Under 35
    - 30-35
    - 35-39
    - 40-44
    - 45+
    - Don’t know
31. Based on your knowledge, how much does cigarette smoking negatively impact female fertility?
    - It doesn’t impact
    - Somewhat impacts
    - Impacts a lot
    - Unsure
32. Based on your knowledge, how much does obesity negatively impact female fertility?
    - It doesn’t impact
    - Somewhat impacts
    - Impacts a lot
    - Unsure
33. How frequently do you think miscarriages occur in Australian women?
    - 1 in 4 women
    - 1 in 8 women
    - 1 in 20 women
    - 1 in 50 women
    - 1 in 100 women
    - Unsure
34. On average, how often do you think IVF and other reproductive technologies achieve a live birth for a patient? IVF refers to in-vitro fertilisation, an assisted reproduction technique where the sperm and egg are collected from the patients and allowed to bind outside of the female body in a specialised laboratory setting
    - 0-25%
    - 26-50%
    - 51-75%
    - 76-100%
    - Unsure
35. Is there something we have missed? Is there anything else you would like to tell us about your experiences using fertility apps, how you access information about your fertility or your knowledge about female reproduction?
