# Supplementary material for: The association between reproductive health smartphone applications and fertility knowledge of Australian women
Source: BMC Womens Health. 2020 Mar 4;20:45. doi: 10.1186/s12905-020-00912-y (PMC7057638; doi:10.1186/s12905-020-00912-y)
Supplement: Supplementary file 2 — Additional file 2: Figure S1. Description of data: Response to fertility knowledge questions by app use status. Each of the questions in the fertility knowledge quiz is displayed with the responses as shown to participants (Δ indicates the correct answer). Black bars represent the number of non-app using respondents (n = 383) that selected a given response, and grey bars indicate app users (n = 290). [file 12905_2020_912_MOESM2_ESM.pptx]

## Slide 1
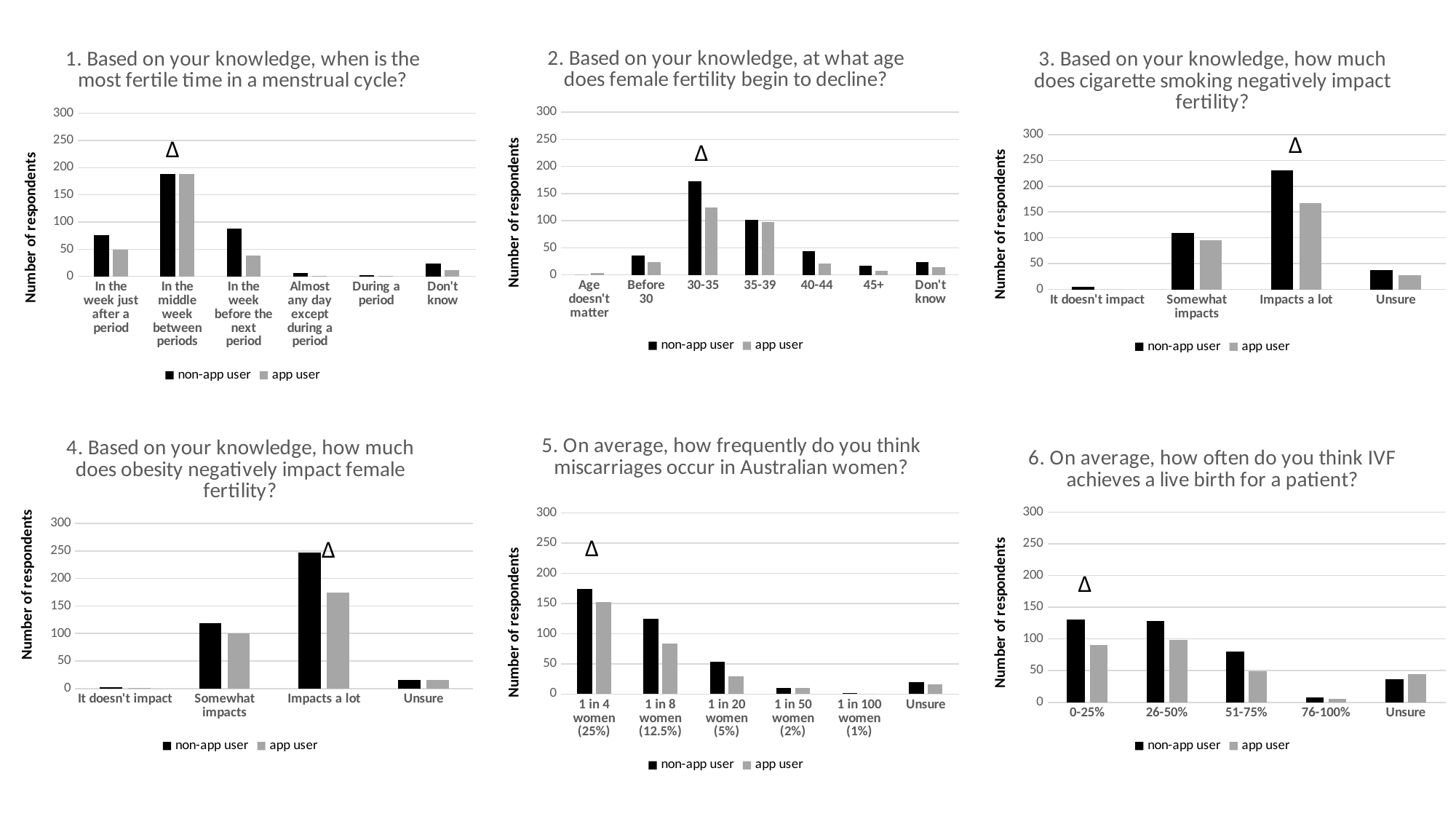

### Chart: 1. Based on your knowledge, when is the most fertile time in a menstrual cycle?
| Category | non-app user | app user |
|---|---|---|
| In the week just after a period | 76.0 | 49.0 |
| In the middle week between periods | 188.0 | 188.0 |
| In the week before the next period | 88.0 | 39.0 |
| Almost any day except during a period | 6.0 | 1.0 |
| During a period | 2.0 | 1.0 |
| Don't know | 23.0 | 12.0 |
### Chart: 2. Based on your knowledge, at what age does female fertility begin to decline?
| Category | non-app user | app user |
|---|---|---|
| Age doesn't matter | 0.0 | 3.0 |
| Before 30 | 36.0 | 23.0 |
| 30-35 | 173.0 | 124.0 |
| 35-39 | 101.0 | 97.0 |
| 40-44 | 44.0 | 21.0 |
| 45+ | 17.0 | 8.0 |
| Don't know | 23.0 | 14.0 |
### Chart: 3. Based on your knowledge, how much does cigarette smoking negatively impact fertility?
| Category | non-app user | app user |
|---|---|---|
| It doesn't impact | 5.0 | 0.0 |
| Somewhat impacts | 110.0 | 95.0 |
| Impacts a lot | 231.0 | 167.0 |
| Unsure | 37.0 | 28.0 |Δ
Δ
Δ
### Chart: 4. Based on your knowledge, how much does obesity negatively impact female fertility?
| Category | non-app user | app user |
|---|---|---|
| It doesn't impact | 2.0 | 1.0 |
| Somewhat impacts | 119.0 | 100.0 |
| Impacts a lot | 247.0 | 174.0 |
| Unsure | 15.0 | 15.0 |
### Chart: 5. On average, how frequently do you think miscarriages occur in Australian women?
| Category | non-app user | app user |
|---|---|---|
| 1 in 4 women (25%) | 174.0 | 152.0 |
| 1 in 8 women (12.5%) | 124.0 | 83.0 |
| 1 in 20 women (5%) | 53.0 | 29.0 |
| 1 in 50 women (2%) | 10.0 | 10.0 |
| 1 in 100 women (1%) | 2.0 | 0.0 |
| Unsure | 20.0 | 16.0 |
### Chart: 6. On average, how often do you think IVF achieves a live birth for a patient?
| Category | non-app user | app user |
|---|---|---|
| 0-25% | 131.0 | 91.0 |
| 26-50% | 128.0 | 99.0 |
| 51-75% | 80.0 | 49.0 |
| 76-100% | 8.0 | 6.0 |
| Unsure | 36.0 | 45.0 |Δ
Δ
Δ
